# Using nanoemulsions of the essential oils of a selection of medicinal plants from Jazan, Saudi Arabia, as a green larvicidal against *Culex pipiens*

**DOI:** 10.1371/journal.pone.0267150

**Published:** 2022-05-23

**Authors:** Hesham A. Mahran

**Affiliations:** 1 Health Informatics Department, College of Public Health and Tropical Medicine, Jazan University, Jazan, Saudi Arabia; 2 Hygiene, Zoonoses and Epidemiology Department, Faculty of Veterinary Medicine, Beni-Suef University, Beni-Suef, Egypt; Taif University, SAUDI ARABIA

## Abstract

Researchers are increasingly looking to plants as sources of novel ingredients active against vector-borne diseases. Medicinal plant extracts and their metabolites are an attractive source for such products. This study investigated the insecticidal activity of five essential oils extracted from the most common medicinal herbs in Jazan province, Kingdom of Saudi Arabia. Extracted oils and nanoemulsions synthesized from those oils were characterized before application at different concentrations to laboratory-reared fourth-stage larvae of Culex pipens. Basil (*Ocimum bascilicum*) and cumin (*Cuminum cyminum*) essential oils showed moderate larvicidal effect with LC50 81.07 ug/mL and 96.29 ug/mL, respectively. That activity was improved in their nanoemulsion forms, as evidenced by a reduction in the LC50 to 65.19 ug/mL for basil and 64.50 ug/mL for cumin. Clove (*Syzygium aromaticum*), henna (*Lawsonia inermis*) and ginger (*Zingiber officinalis*) oils showed weaker insecticidal activity, with LC50 values of 394 ug/mL, 306 ug/mL, and 494 ug/mL, respectively. Moreover, the nanoemulsion forms of those essential oils did not show any improvement in their insecticidal activity. In conclusion, of the studied plants, the nanoemulsions of basil and cumin showed significant larvicidal activity.

## Introduction

The Jazan region is located along 300 km of the Red Sea coast in the southern part of the Kingdom of Saudi Arabia. It is endowed with a wealth of medicinal plants that the people of the region have used in traditional medicine for centuries, not only in the local area but also worldwide. Furthermore, these medicinal herbs have now become a major source for new medicines and drugs [1]. Clove oil (*Syzygium aromaticum*), for example, can be used to treat dental pain, and ginger root (*Zingiber officinalis*) can be used for colic and stomach pain [2]. A common traditional use of medicinal plants in Jazan Province is against the effects of mosquito bites. There are seven genera and sixteen species of mosquitoes in the province [3] associated with diseases such as Dengue Fever, which can be transmitted by *Aedes aegypti* [4], and skin allergies, caused by the common house mosquito (*Culex pipiens*) [5]. Garlic (*Allium sativum*), basil (*Ocimum bascilicum*), mint (*Mentha piperita*), sage (*Salvia officinalis*), and lemongrass (*Cymbopogon citratus*) have all been used by local people as insect or mosquito repellents. These treatments may lack the toxic side-effects to humans of chemical insecticides, such as skin allergies, respiratory failure, gastrointestinal disturbances, muscle tremors, seizures, paralysis, and death [6].

Despite there being, by some estimates, 141 species of medicinal plants and herbs in the Jazan region, there is currently a lack of research to estimate or assess their effectiveness in a range of potential treatments, including as insect or mosquito repellents [7].

To fill this gap, we tried in this study to evaluate the use of five of these herbs, namely cumin (*Cuminum cyminum*), basil (*Ocimum bascilicum*), henna (*Lawsonia inermis*), ginger (*Zingiber officinal*), and clove oil (*Syzygium aromaticum*) against the common house mosquito (*Culex pipiens*) [8, 9]. Convenient laboratory methods were used to prepare and test the ingredients. The produced data was analyzed statistically to a significance level of an Alpha-value of less than 0.05 [10].

A particular feature of this study is the preparation of essential oils from the selected herbs into nanoemulsions in order to compare the effectiveness against mosquitos of those nanoemulsions with that of conventional essential oils. Nanotechnology is considered an excellent alternative technique for drug delivery system development and research, and offers the potential to deliver drugs more directly to the intended place of action, with associated increased therapeutic efficacy, lower doses and lower dose frequencies needed for the same clinical effect, and reduced incidence and severity of side effects. Nano-systems can also protect the active substances against many degradation and inactivation mechanisms. Moreover, they can enhance the degree of incorporation between substances with different polarities in relation to the matrix, which can serve to support prolonged drug-release, and drug action into a particular tissue. These benefits arise from various factors. For example, the larger surface contact area with the targeted tissue can increase distribution, absorption, and drug uptake. Also, the physicochemical properties of the coating or matrix polymers can supply better protection against target organisms’ physiological barriers, or offer more affinity with the target tissue [11, 12].

Regarding mosquito control strategies, larval control should be considered as the first option for diminution [13]. This involves control in larval habitat locations, such as stagnant pools after rainfall, which are considered the main breeding sites in the Jazan area. If mosquito larvae in the suspected breeding sites can be controlled, the adults may never develop and the larvae are concentrated, immobile, and reachable. Hence, larvicidal control has the most significant impact in efforts to control mosquito populations [14] and thereby improve public and environmental health.

## Materials and methods

### 1. Source and preparation of essential oils

The essential oils used in this work—*Ocimum bascilicum* (basil), *Cuminum cyminum*, *Syzygium aromaticum* (clove), *Lawsonia inermis* (henna), and *Zingiber officinalis* (ginger)—were obtained from local sources in Jazan. Seven concentrations (2000, 1000, 500, 250, 125, and 62.50 ug/mL) of *Lawsonia inermis* (Henna) *Zingiber officinalis* (ginger) and *Syzygium aromaticum* (clove) were prepared by dissolving the oils in 70% ethanol. Meanwhile, six concentrations (1000, 500, 250, 125, 62.50, and 31.25 ug/mL) of *Cuminum cyminum* and *Ocimum bascilicum* were prepared by the same method [15].

### 2. GS C-MSS of essential oils

The analysis of all the essential oils was conducted at the Nawah Scientific Educational Research Center, Egypt (https://nawah-scientific.com/) using gas chromatography-mass spectrometry (GC-MS). The GC-MS analyses were carried out using TRACE GC Ultra Gas Chromatographs (THERMO Scientific Corp., USA) [16], coupled with a Thermo mass spectrometer detector (ISQ Single Quadrupole Mass Spectrometer). The GC-MS system was equipped with a TR-5 MS column (30 m x 0.32 mm i.d., 0.25 μm film thickness). Analyses were carried out using helium as a carrier gas at a flow rate of 1.0 mL/min and a split ratio of 1:10 and with the following temperature program: 60°C for 1 min; rising at 4.0°C/min to 240°C and the held for 1 min. The injector and detector were held at 210°C. Diluted samples (1:10 hexane, v/v) of 1 μL of the mixtures were always injected. Mass spectra were obtained by electron ionization (EI) at 70 eV, using a spectral range of m/z 40–450. The identification of the chemical constituents of the essential oil was de-convoluted using AMDIS software (www.amdis.net) and identified by retention indices (relative to n-alkanes C8-C22), mass spectrum matching to (authentic standards (when available) from the Wiley spectral library collection and NSIT library database.

### 3. Preparation of nanoemulsions of the essential oils

Briefly, macroemulsions of oil/water were prepared by combining the essential oils with Tween 80 as a surfactant (one oil to three T80); then adding water to obtain a concentration of 2.50%, mixing by using a magnetic stirrer (with a speed of 500 rpm, for 10 min). The prepared macroemulsion was then sonicated for 5 min using an ultrasonicator (750 W, Branson Probe sonicator-Advanced model, 20 kHz). The resulting nanoemulsions were characterized by a UV-visible spectrophotometer (UV-2600, **Shimadz**, Japan) at 345 nm [17].

### 4. Characterization of nanoemulsions of the used essential oils

The droplet size distribution (d, nm) (analysis by volume) and polydispersity index (PDI) of nanoemulsions were measured by a zeta sizer apparatus (dynamic light scattering technique) (Nano-ZS90, Malvern, UK). Prior to the experiment, all the samples were diluted to 10% with deionized water in order to reduce scattering effects [18].

### 5. Preparation of *C*. *pipiens* larvae

Egg rafts of a laboratory-reared colony of *C*. *pipiens* were sieved into convenient plastic containers with water. Then, the resulted larvae were placed in enamel trays with one liter of dechlorinated water and 0.15 grams of Brewer’s yeast (lactalbumin) (50:50). Water was replaced every other day, and food was added on a daily basis. Adults were kept in 0.51 m^3^ aluminum screen cages and fed on 10% sucrose solution on cotton wicks. A restrained quail was used to blood-feed the insect female. A 400 ml plastic container was used to collect the deposited egg rafts. The colony was kept at 26°C and at 75% RH with a 16 L: 8 D photoperiod. In the bioassays, the third and fourth larval instars were used [19].

### 6. Larvicidal bioassay against *Culex pipiens*

The standard method of the World Health Organization was used in this bioassay [20]. This was done in plastic cups (250 mL). The essential oils were dissolved in ethyl alcohol at the tested concentrations (0.312, 0.625, 1.25, 2.5, 5.0, and 10.0%) then the working solution prepared by aliquot: one mL of these dilutions were added to 99 mL distilled water. Twenty *Culex pipiens* third-instar larvae were placed in the prepared concentrations in the plastic cups (five replicates for each concentration). In the negative control, larvae were exposed to one mL of the solvent dissolved in the water. After 24 h, dead larvae (motionless) were recorded, and the average percentage mortality was estimated [21]

### 7. Statistical analysis

Five replicates were done for all the treatments, and mean ± SE values were calculated. Larval mortality analysis was performed by using ANOVA and subsequent Duncan’s multiple range tests (p < 0.05). Also, Probit analysis was applied to determine the LC_50_ and LC_90_ values with their 95% confidence limits [22]. All statistical tests and analyses were achieved using SPSS (IBM SPSS Version 22.0).

## Results

### GC-Ms analysis of the medicinal plants’ essential oils

The GC-MS analysis of basil oil analysis demonstrated many components, but those with the highest concentrations were linalool, eucalyptol, and eugenol at 20, 12, and 7.91%, respectively (S1 Table). The analysis of cumin revealed a composition of 29.29% benzaldehyde, 4-(1-methylethyl), 17.1% à-Pinene and 15.42% 2-CAREN-10-AL (S2 Table). Clove oil contained 10.29% heptadecane, 11.34% Caryophyllene, 7.93% Hexadecane, 7.92% docosane, 7.91% dotriaconate, and 7.68% octadecane (S3 Table). Henna extract revealed 92.13% oleic acid, 5.42% n-Hexadecanoic acid, and 2.45% Hexadecanoic acid, methyl ester (S4 Table). The main components of ginger oil were 9,12-Octadecadienoic acid (Z,Z)- (23.83%), zingiberene (18.56%), α-curcumene (14.58%), á-sesquiphellandrene (12.85%), and á-Bisabolene (11.46%) (S5 Table).

### Characterization of the nanoemulsions prepared from the essential oils

**Fig 1:**
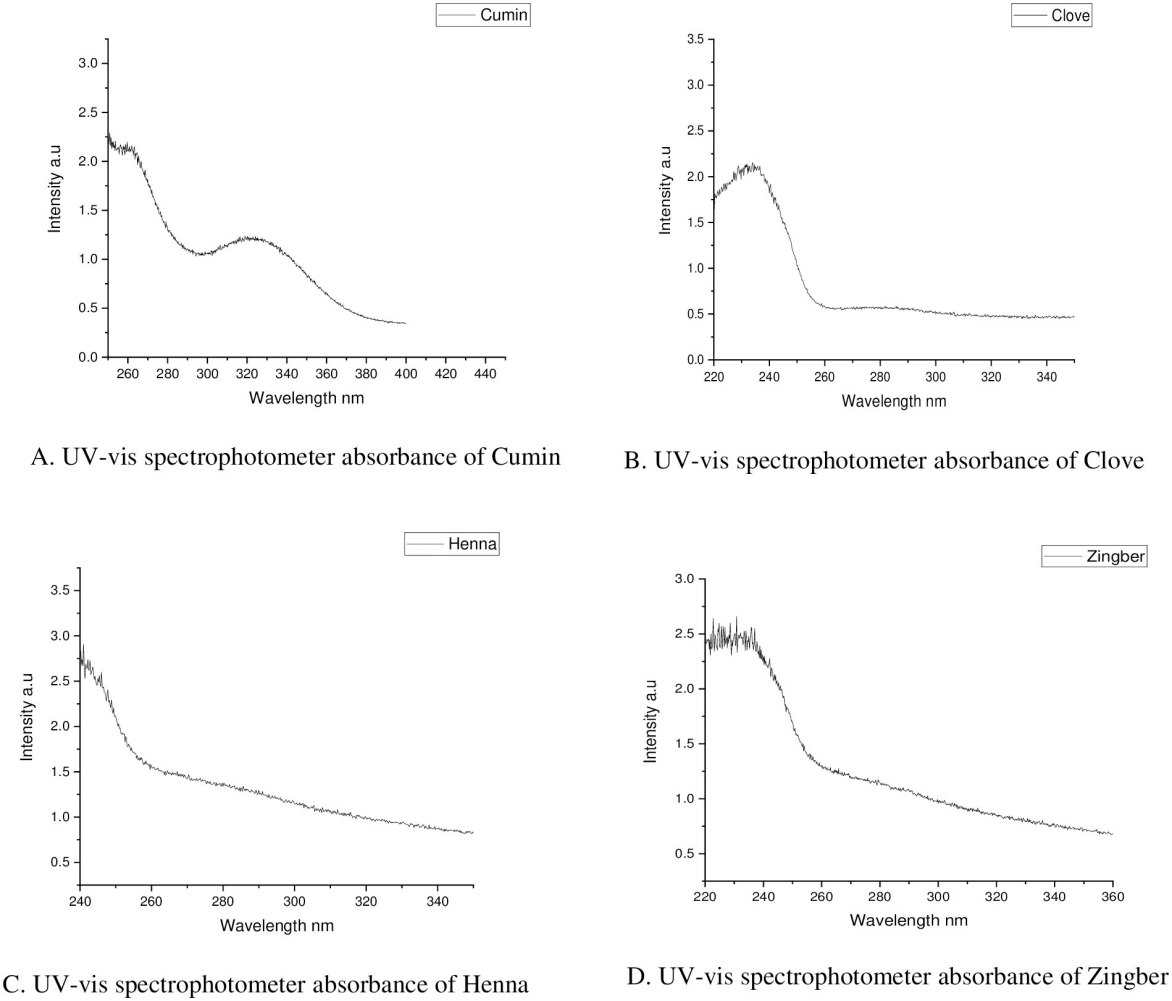
UV-vis spectrophotometer absorbance of different essential oils nanoemulsions.

**Fig 2:**
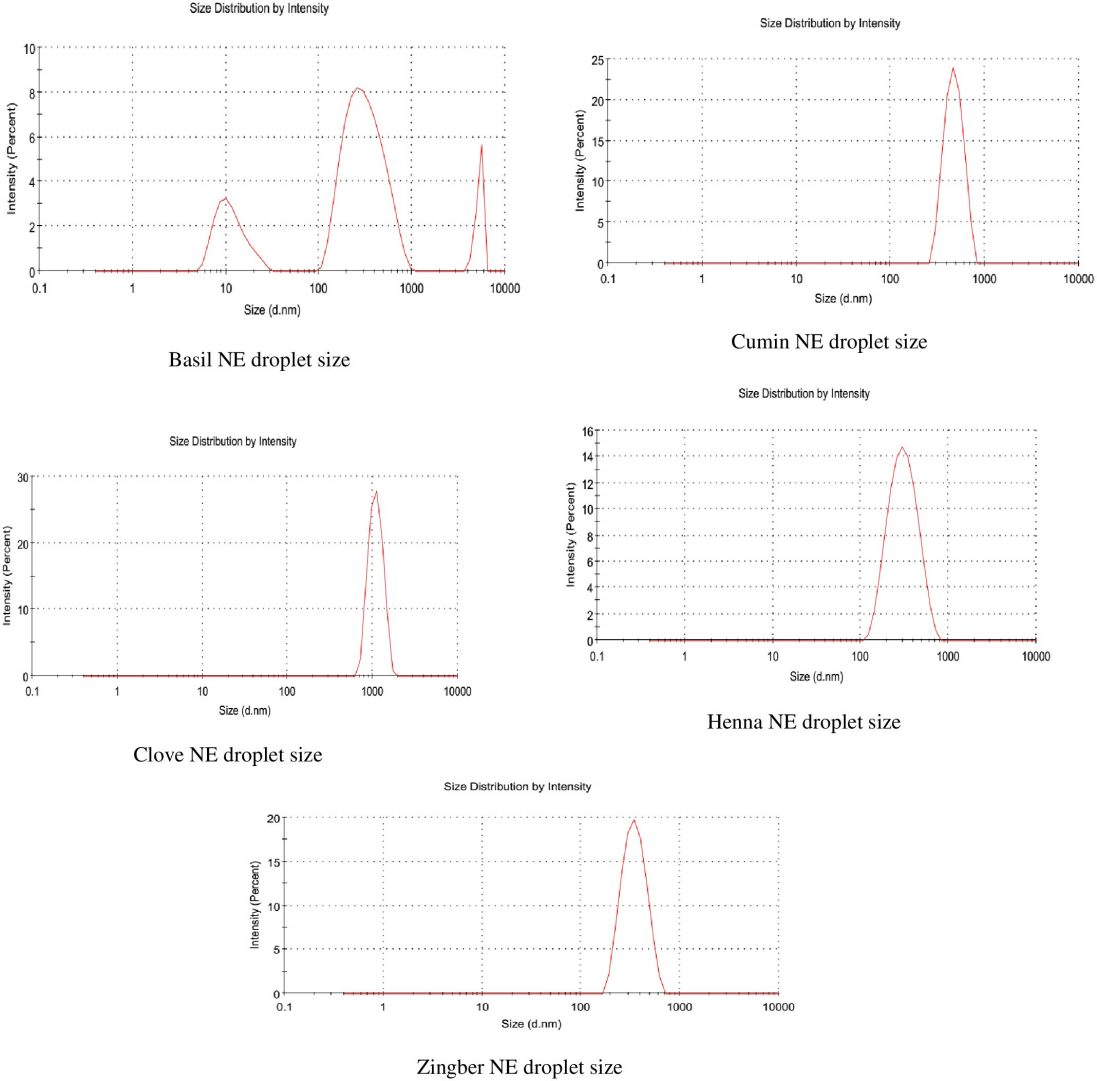
Droplet size of different essential oils nanoemulsions.

The hydrodynamic particle size of basil nanoemulsion was 215 nm with PDI 0.729. For cumin, the droplet size (DS) was 446.2 nm with PDI 0.047. The droplet size of clove nanoemulsion was 996.4 nm with PDI 0.224. The henna nanoemulsion droplet size was 273.9 with PDI 0.143. The droplet size of ginger nanoemulsion was 325.1 nm with PDI 0.059 (Table 1). The low PDI values reflected the homogenous size distribution of droplets in each essential nanoemulsion (Figs 1 & 2).

**Table 1:** Zeta sizer of the nanoemulsion of each essential oil.

Eos	Droplet size (nm)	PDI*
Basil	215	0.729
Cumin	446.2	0.047
Clove	996.4	0.224
Henna	273.9	0.143
Ginger	325.1	0.059

* PDI = polydispersity index of nanoemulsions

### Larvicidal activity of the used essential oils and their nanoemulsion forms

Regarding the larvicidal potency of basil essential oils, the LC_50_ was 81.07 ug/mL and LC_90_ was 227.9ug/mL. The basil nanoemulsion, meanwhile, showed an LC_50_ of 65.19 ug/mL, and an LC_90_ of 193.3 ug/mL (Table 2). For cumin essential oils, it was 96.29 ug/mL and 64.50 ug/mL, respectively (Table 2). It was observed that in both of these cases the LC_50_ and LC_90_ values of the nanoemulsion forms were lower than the ordinary form. Turning to clove, the LC_50_ was 394 ug/mL, and LC_90_ was 1658 ug/mL, with henna the LC_50_ was 306 ug/mL and the LC_90_ was 1703 ug/mL for (Table 2). Ginger showed an LC_50_ and LC_90_ of 494 ug/mL and 1682 ug/mL, respectively. Moreover, it is noteworthy that the larvicidal effect of the essential oils and their nanoemulsion forms was concentration dependent. Basil and cumin essential oils achieved 100% larvicidal mortality at concentrations of 250, 500 and 1000 ug/mL. This insecticidal activity decreased to reach its minimal effect at the lowest concentration 31.25 ug/mL (Tables 3 & 4). In the case of clove, henna and ginger, however, the nanoemulsion had no additional effect beyond that of the essential oils (Table 2). Clove and ginger essential oils showed only weak larvicidal effects, such that only the highest concentration, 2000 ug/mL, caused 100% mortality of larvae (Tables 5 & 6). For henna, meanwhile, concentrations of 1000 and 2000 ug/mL caused 100% mortality of larvae (Table 7).

**Table 2:** LC50 and LC95 of the used essential oils after 24 hours of application.

Treatments	LC50 (95 CI)	LC95 (95 CI)	Slope±SE
Basil ordinary form	81.07 (64.2–102.6)	227.9 (163.3–430.03)	3.6±0.6
Basil nanoemulsion	65.19 (50.5–82.7)	193.3(137.2–378.7)	3.48±0.6
Cuminum ordinary form	96.29 (77.7–120.7)	242.36(177.8–434.8)	4.1±0.7
Cuminum nanoemulsion	64.5(48.3–83.4)	222.9(151.5–489.09)	3.05±0.6
Clove ordinary form	394(111–2096)	1658(651–4882348)	2.6±0.3
Clove nanoemulsion	379(198–810)	1815(838–19457)	2.4±0.3
Henna ordinary form	306(168–718)	1703(724–31076)	2.2±0.3
Henna nanoemulsion	288(153–712)	1869(742–47639)	2.02±0.3
Ginger ordinary form	494(388–636)	1682(1171–3117)	3.9±0.4
Ginger nanoemulsion	479(376–615)	1623(1134–2935)	3.1±0.4

**Table 3:** Mean of larval mortalities after treatment with basil and its emulsion form after 24 hours application on the fourth larval stage of laboratory-reared *Culex pipiens*.

Form Treatment	Basil Mean ± Std. Error	Emulsion Basil Mean ± Std. Error
Control untreated	4.80±.37^a^	4.80±.37^a^
Deltamethrin (0.755ppm)	100.0±.00^h^	100.0±.00^h^
1000 ug/mL	100.00±.00^h^	100.00±.00^h^
500 ug/mL	100.00±.00^h^	100.00±.00^h^
250 ug/mL	100.00±.00^h^	100.00±.00^h^
125 ug/mL	71.20±.37^f^	87.40±.24^g^
62.5 ug/mL	29.80±.37^d^	37.80±.37^e^
31.25 ug/mL	12.40±.24^b^	19.80±.37^c^

Superscript of the same letter in cells of the same column shows a non-significant effect in respect to the non-treated control. Superscript of different letters in cells of the same column shows a significant effect in respect to the non-treated control (*P* ≤ 0.05).

**Table 4:** Mean of larval mortalities after treatment with cumin and its emulsion form after 24 hours application on the fourth larval stage of laboratory-reared *Culex pipiens*.

Form Treatment	*Cuminum cyminum* Mean ± Std. Error	Emulsion Mean ± Std. Error
Control untreated	4.80 ±.37^a^	4.80 ±.37^a^
Deltamethrin (0.755ppm)	100.0±.00^h^	100.0±.00^h^
2000 ug	100.00±.00^h^	100.00±.00^h^
1000 ug/mL	100.00±.00^h^	100.00±.00^h^
500 ug/mL	100.00±.00^h^	100.00±.00^h^
250 ug/mL	100.0±.00^h^	100.0±.00^h^
125 ug/mL	60.00±.44^d^	84.80±.37^e^
62.5 ug/mL	20.00±.31^b^	34.80±.58^c^
31.2 ug/mL	8.80±.37^a^	22.40±.40^b^

Superscript of the same letter in cells of the same column shows a non-significant effect in respect to the non-treated control. Superscript of different letters in cells of the same column shows a significant effect in respect to the non-treated control (*P* ≤ 0.05).

**Table 5:** Mean of larval mortalities after treatment with clove and its emulsion form after 24 hours application on the fourth larval stage of laboratory-reared *Culex pipiens*.

Form Treatment	Clove Mean Std. Error	Emulsion Mean Std. Error
Control untreated	4.80±.37^a^	4.80±.37^a^
Deltamethrin (0.755ppm)	100.00±.00^e^	100.00±.00^e^
2000 ug	100.00±.00e	100.00±.00^e^
1000 ug/mL	89.80±.37^d^	90.00±.31^d^
500 ug/mL	54.80±.37^c^	54.80±.37^c^
250 ug/mL	24.80±.37^b^	26.20±.37^b^
125 ug/mL	7.40±.40^a^	8.40±.40^a^
62.5 ug/mL	3.60±.24^a^	3.80±.20^a^
31.2 ug/mL	2.60±.24^a^	3.80±.20^a^

Superscript of the same letter in cells of the same column shows a non-significant effect in respect to the non-treated control. Superscript of different letters in cells of the same column shows a significant effect in respect to the non-treated control (*P* ≤ 0.05).

**Table 6:** Mean of larval mortalities after treatment with ginger and its emulsion form after 24 hours application on the fourth larval stage of laboratory-reared *Culex pipiens*.

Form Treatment	Ginger Mean Std. Error	Emulsion Ginger Mean Std. Error
Control untreated	4.80±.37^a^	4.80±.37^a^
Deltamethrin (0.755ppm)	100.00±.00^e^	100.00±.00^e^
2000 ug	100.00±.00e	100.00±.00^e^
1000 ug/mL	88.60±.50^d^	90.00±.54^d^
500 ug/mL	49.80±.37^c^	50.00±.31^c^
250 ug/mL	22.40±.50^b^	24.80±.37^b^
125 ug/mL	8.60±.24^a^	9.80±.20^a^
62.5 ug/mL	3.60±.24^a^	4.80±.20^a^
31.2 ug/mL	3.60±.24^a^	4.80±.20^a^

Superscript of the same letter in cells of the same column shows a non-significant effect in respect to the non-treated control. Superscript of different letters in cells of the same column shows a significant effect in respect to the non-treated control (*P* ≤ 0.05).

**Table 7:** Mean of larval mortalities after treatment with henna and its emulsion form after 24 hours application on the fourth larval stage of laboratory-reared *Culex pipiens*.

Form Treatment	Henna Mean ±S.E	Emulsion Mean ±S.E
Control untreated	4.80±.37^a^	4.80±.37^a^
Deltamethrin (0.755ppm)	100.00±.00^e^	100.00±.00^e^
2000 ug	100.00±.00e	100.00±.00^e^
1000 ug/mL	100.00±.00^e^	100.00±.00^e^
500 ug/mL	64.80±.37^d^	65.00±.32^d^
250 ug/mL	20.00±.32^c^	26.80±.37^c^
125 ug/mL	15.20±.20^b^	19.80±.20^b^
62.5 ug/mL	6.20±.37^a^	6.60±.51^a^
31.2 ug/mL	3.60±.24^a^	4.80±.20^a^

Superscript of the same letter in cells of the same column shows a non-significant effect in respect to the non-treated control. Superscript of different letters in cells of the same column shows a significant effect in respect to the non-treated control (*P* ≤ 0.05).

## Discussion

Although more than 141 kinds of medicinal plants and herbs are thought to grow in the Jazan region, little research has been conducted to assess their usefulness [8]. In this paper, we try to fill this gap in respect to the larvicidal effects of some of these herbs, particularly basil (*Ocimum bascilicum*), cumin (*Cuminum cyminum*), clove (*Syzygium aromaticum*), henna (*Lawsonia inermis*), and ginger (*Zingiber officinalis*). Specifically, we use convenient laboratory methods for the preparation and nano-emulsification of their essential oils and then test for larvicidal effects against the common house mosquito (*Culex pipiens*). We found that basil (*Ocimum bascilicum*) from the Jazan region contains several constituents, mainly linalool (20.6%), eucalypol (12.45%), and eugenol (7.91%). This contrasts with northwest Iran, where eugenol has been found to be the principal component in basil (19.22%), followed by linalool (12.63%), and with only a small proportion of eucalyptol (1.79%) [23]. Also, in Fiji, basil contains mainly linalool (22.3%) and methyleugenol (24.7%), while, in Cuba, it contains mainly methylchavicol (66.8%), 1,8-cineole (5.4%) and linalool (5.0%). In Burkina Faso, it contains mainly: 1,8-cineole (60.2%), α-terpineol (6.5%) and β-pinene (5.7%) [14]. These percentages may vary according to many factors, such as the place of cultivation and method of extraction.

Cumin (*Cuminum cyminum*) in Jazan contains mainly Benzaldehyde, 4-(1-methylethyl) (29.39%), à-Pinene (17.1%), and 2-Caren-10-al (15.42%). Tunisian Cuminum cyminum contains mainly cuminlaldehyde (39.48%), gamma-terpinene (15.21%), O-cymene (11.82%), and beta-pinene (11.13%) [23, 24], characterized γ-terpinen-7-al (35.3%), and cumin aldehyde (21.8%) in cumin essential oils. These variations are due to many factors, including the place of cultivation, method of extraction, and time of harvesting [25, 26].

Clove oil (*Syzygium aromaticum*) contains mainly caryophyllene (11.34%), heptadecane (10.29%), hexadecane (7.93%), docosane (7.92%), and a low percentage of eugenol (3.98%). The eugenol content of clove oil has previously been reported to vary greatly and may reach up to (72–90%), [27] or even 99.10% [28] according to the country of origin. Our sample contains a very low eugenol content which may refer to place of cultivation, method of extraction, part of the plant used in extraction, and harvest time [29].

Henna (*Lawsonia inermis*) contains mainly oleic acid (92.13%), n-hexadecanoic acid (5.42%), and hexadecanoic acid, methyl ester (2.45%). Again, these percentages vary according to many factors [30], including place of cultivation and cultivation condition. The chemical composition of *Lawsonia inermis*, in Sudan [31], Nigeria, Egypt [32] and also in Ethiopia have shown a composition of 17.61% eugenol, 15.07% hexadecanoic acid and 10.17% phytol [33].

Ginger (*Zingiber officinalis*) contains several constituents, mainly: 9,12-Octadecadienoic acid (23.83%), Zingiberene (18.56%), and α-Curcumene (14.58%). The main phenolic compounds in ginger are gingerols (the major polyphenols), shogaols, and paradols [34], as well as quercetin, gingerene-A, zingerone, and 6-dehydrogingerdine [35]. There are also many terpene components, which are considered to be the main constituents of ginger essential oils, such as β-bisabolene, α-farnesene, α-curcumene, zingiberene, and β-sesquiphellandrene [36].

Regarding the insecticidal effect of these essential oils, our results showed that the lethal concentrations (LCs) of basil essential oils are LC_50_ 81.07 μg/mL and LC_95_ 227.9 μg/mL. These essential oils have previously been shown to exhibit a repellant activity against insects, and larvicidal activity against blue bottle flies, houseflies, and mosquitoes. Specifically, [37, 38] found the LC_50_ and LC_90_ values of basil were, respectively, 73.45 and 101.20 ppm against fourth instar larva of *C*. *pipens*. Application of *Ocimum basilicum* essential oil for 24 hours revealed moderate LC_50_ and LC_90_ values of 141.95 ppm and 100.82 ppm, respectively [39]. The essential oils of *Ocimum basilicum* have repellant activities against female Anopheles, and could be used in the form of natural repellent cream [40].

For cumin essential oils, LC_50_ in this study was 96.29 ug/mL, and LC_90_ was 222.9 ug/mL. This finding differs from a previous study in which the methanolic extract of *C*. *cyminum* seed showed 87.20 ±1.92% mortality against *An*. *stephensi* (LC_50_ = 500 ppm), and 75.60 ± 2.48% against *Culex*. *Quinquefasciatus* at a dose of 500 ppm [41]. Cumin has also been shown to exhibit significant action on adults of *Musca persicae* (LC50 = 3.2 ml L− 1) and Musca domestica (LD50 = 31.8 μg adult1) [42].

The recorded LC_50_ of clove was 394 ug/mL, and the LC_90_ was 1658 ug/mL. These results differ from another trial that has reported LC_50_ and LC_95_ values of 17.527 ug/mL and 2374.06 ug/mL for clove against Anopheles gambiae larvae [43]. Also, many previous studies have demonstrated the use of these ordinary forms for purposes such as insecticides against herbal worms [44].

The LC_50_ of henna essential oil in this study was 306 ug/mL and the LC_90_ was 1703 ug/mL. Previously, the broth extract of the *Lawsonia inermis* leaves has been found to demonstrate an obvious antibacterial activity against *E*. *coli* [45]. The phytochemical compounds of henna have antibacterial and antioxidant activity [46], while the methanolic extract of *Lawsonia inermis* has been found to be effective against nymphs and adults of the cotton mealybug Phenacoccus solenopsis Tinsley (Hemiptera: Pseudococcidae) [47]. The insecticidal activity of fruit and leaves of *Lawsonia inermis* has also been estimated against the major pest of stored grains, the red flour beetle, *Tribolium castaneum* (Herbst) [48].

*Zingiber officinalis* showed LC_50_ and LC_90_ of 494 ug/mL and 1682 ug/mL, respectively. It showed 45% insecticidal activity against *Culex theileri* [49]. Also, *Z*. *cassumunar* essential oil demonstrated moderate larvicidal activity against first instar larvae of *Aedes albopictus* with an LC_50_ of 44.9 mg/L after 24 h [50].

The present study has added to previous work by showing that the nanoemulsion form of basil essential oil exhibited a lower LC_50_ than the ordinary essential oil. Specifically, the nanoemulsion form had an LC_50_ 19.58% (81.07–65.19 = 15.88) lower than that of the essential oil. This demonstrates that the nanoemulsion form of *O*. *basilicum* essential oil is more potent against the common house mosquito (*Culex pipiens*). This finding supports other work suggest that nanoemulsion forms improve the larvicidal [51, 52] and insecticidal activities of essential oils [53]. Similarly, the nanoemulsion form of cumin essential oil showed a 33.01% (96.29–64.5 = 31.79) lower LC_50_ than that of the ordinary form. This result is supported by other studies that have used these ordinary and nanoemulsion forms for a range of purposes, including as insecticides [42].

In contrast, the nanoemulsion forms of clove, henna (*Lawsonia inermis*) and ginger (*Zingiber officinalis*) essential oils showed the same LC50 value as the ordinary form, meaning that there was no increased potency arising from the nanoemulsion forms of these essential oils.

The improved insecticidal activity of basil and cumin nanoemulsions may arise from inherent physicochemical properties [54]. This nanometer size of the essential oil nanoemulsions has been suggested to improve its delivery targeting and specificity, which reflects on increasing its effective nanoemulsions than crude nanoemulsion. Furthermore, nanoemulsions have a low concentration of surfactants, which reduces both economic and environmental costs [55]. Moreover, the nano-emulsification is recognized as supporting better drug delivery [57] and better pesticide [56] and biopesticide formulations [57]. Essential oil nano-formulations can inhibit enzymatic function in insects and can cause growth hormone deregulation, ultimately stopping the insect shedding, which leads to insect death [58, 59]. Moreover, basil nanoemulsion has potent larvicidal activity against Culex quinquefasciatus [59].

In summary, this study reveals that basil (*Ocimum bascilicum*) and cumin (*Cuminum cyminum*) essential oils gathered from the Jazan region of Saudi Arabia demonstrated significant activity against the common house mosquito (*Culex pipiens*), but also that their nanoemulsion forms showed a greater effectiveness than the ordinary forms. In contrast, clove (from *Syzygium aromaticum*), henna (*Lawsonia inermis*) and ginger (*Zingiber officinalis*) essential oils showed less (non-significant) activity, and the nanoemulsion forms of these essential oils also did not improve their insecticidal activity.

## Supporting information

S1 Data (DOCX)

S1 Table The phytochemical composition of basil by GC-MS. (DOCX)

S2 Table The phytochemical composition of cumin by GC-MS. (DOCX)

S3 Table The phytochemical composition of clove by GC-MS. (DOCX)

S4 Table The phytochemical composition of henna by GC-MS. (DOCX)

S5 Table The phytochemical composition of zingber by GC-MS. (DOCX)
