# Supplementary material for: Using nanoemulsions of the essential oils of a selection of medicinal plants from Jazan, Saudi Arabia, as a green larvicidal against Culex pipiens
Source: PLoS One. 2022 May 23;17(5):e0267150. doi: 10.1371/journal.pone.0267150 (PMC9126372; doi:10.1371/journal.pone.0267150)
Supplement: S1 Data — (DOCX) [file pone.0267150.s001.docx]

**Using Nanoemulsions of the Essential Oils of a Selection of Medicinal Plants from Jazan, Saudi Arabia, as a Green Larvicidal against *Culex pipiens***

***Hesham A. Mahran^1,2^***

***^1^***Health Informatics Department,College of Public Health and Tropical Medicine, Jazan University, Jazan, Saudi Arabia, ***^2^***Hygiene, Zoonoses and Epidemiology Department, Faculty of Veterinary Medicine, Beni-Suef University, 52611, Beni-Suef, Egypt.

**Abstract**

Researchers are increasingly looking to plants as sources of novel ingredients active against vector-borne diseases. Medicinal plant extracts and their metabolites are an attractive source for such products. This study investigated the insecticidal activity of five essential oils extracted from the most common medicinal herbs in Jazan province, Kingdom of Saudi Arabia. Extracted oils and nanoemulsions synthesized from those oils were characterized before application at different concentrations to laboratory-reared fourth-stage larvae of Culex pipens. Basil (*Ocimum bascilicum*) and cumin (*Cuminum cyminum*) essential oils showed moderate larvicidal effect with LC50 81.07 ug/mL and 96.29 ug/mL, respectively. That activity was improved in their nanoemulsion forms, as evidenced by a reduction in the LC50 to 65.19 ug/mL for basil and 64.50 ug/mL for cumin. Clove (*Syzygium aromaticum*), henna (*Lawsonia inermis*) and ginger (*Zingiber officinalis*) oils showed weaker insecticidal activity, with LC50 values of 394 ug/mL, 306 ug/mL, and 494 ug/mL, respectively. Moreover, the nanoemulsion forms of those essential oils did not show any improvement in their insecticidal activity. In conclusion, of the studied plants, the nanoemulsions of basil and cumin showed significant larvicidal activity.

**Keywords:** Jazan, Nano-emulsion, medicinal plants, Larvicidal, Culex pipiens

**Materials and Methods**

**1. Source and preparation of essential oils**

The essential oils used in this work—*Ocimum bascilicum* (basil)*, Cuminum cyminum, Syzygium aromaticum* (clove), *Lawsonia inermis* (henna), and *Zingiber officinalis* (ginger)—were obtained from local sources in Jazan. Seven concentrations (2000, 1000, 500, 250, 125, and 62.50 ug/mL) of *Lawsonia inermis* (Henna) *Zingiber officinalis* (ginger) and *Syzygium aromaticum* (clove) were prepared by dissolving the oils in 70 % ethanol. Meanwhile, six concentrations (1000, 500, 250, 125, 62.50, and 31.25 ug/mL) of *Cuminum cyminum* and *Ocimum bascilicum* were prepared by the same method. [15]

**2. GS C-MSS of essential oils**

The analysis of all the essential oils was conducted at the Nawah Scientific Educational Research Center, Egypt (<https://nawah-scientific.com/>) using gas chromatography-mass spectrometry (GC-MS). The GC-MS analyses were carried out using TRACE GC Ultra Gas Chromatographs (THERMO Scientific Corp., USA) [16], coupled with a Thermo mass spectrometer detector (ISQ Single Quadrupole Mass Spectrometer). The GC-MS system was equipped with a TR-5 MS column (30 m x 0.32 mm i.d., 0.25 μm film thickness). Analyses were carried out using helium as a carrier gas at a flow rate of 1.0 mL/min and a split ratio of 1:10 and with the following temperature program: 60 °C for 1 min; rising at 4.0 °C/min to 240 °C and the held for 1 min. The injector and detector were held at 210 °C. Diluted samples (1:10 hexane, v/v) of 1 μL of the mixtures were always injected. Mass spectra were obtained by electron ionization (EI) at 70 eV, using a spectral range of m/z 40–450. The identification of the chemical constituents of the essential oil was de-convoluted using AMDIS software (www.amdis.net) and identified by retention indices (relative to n-alkanes C8-C22), mass spectrum matching to (authentic standards (when available) from the Wiley spectral library collection and NSIT library database.

**3. Preparation of nanoemulsions of the essential oils**

Briefly, macroemulsions of oil/water were prepared by combining the essential oils with Tween 80 as a surfactant (one oil to three T80); then adding water to obtain a concentration of 2.50%, mixing by using a magnetic stirrer (with a speed of 500 rpm, for 10 min). The prepared macroemulsion was then sonicated for 5 min using an ultrasonicator (750 W, Branson Probe sonicator-Advanced model, 20 kHz). The resulting nanoemulsions were characterized by a UV-visible spectrophotometer (UV-2600, **Shimadz**, Japan) at 345 nm. [17]

**4. Characterization of nanoemulsions of the used essential oils**

The droplet size distribution (d, nm) (analysis by volume) and polydispersity index (PDI) of nanoemulsions were measured by a zeta sizer apparatus (dynamic light scattering technique) (Nano-ZS90, Malvern, UK). Prior to the experiment, all the samples were diluted to 10% with deionized water in order to reduce scattering effects [18].

**5. Preparation of *C. pipiens* larvae**

Egg rafts of a laboratory-reared colony of *C. pipiens* were sieved into convenient plastic containers with water. Then, the resulted larvae were placed in enamel trays with one liter of dechlorinated water and 0.15 grams of Brewer's yeast (lactalbumin) (50:50). Water was replaced every other day, and food was added on a daily basis. Adults were kept in 0.51 m^3^ aluminum screen cages and fed on 10% sucrose solution on cotton wicks. A restrained quail was used to blood-feed the insect female. A 400 ml plastic container was used to collect the deposited egg rafts. The colony was kept at 26 °C and at 75% RH with a 16 L: 8 D photoperiod. In the bioassays, the third and fourth larval instars were used [19].

**6. Larvicidal bioassay against** *Culex pipiens*

The standard method of the World Health Organization was used in this bioassay [20]. This was done in plastic cups (250 mL). The essential oils were dissolved in ethyl alcohol at the tested concentrations (0.312, 0.625, 1.25, 2.5, 5.0, and 10.0%) then the working solution prepared by aliquot: one mL of these dilutions were added to 99 mL distilled water. Twenty *Culex pipiens* third-instar larvae were placed in the prepared concentrations in the plastic cups (five replicates for each concentration). In the negative control, larvae were exposed to one mL of the solvent dissolved in the water. After 24 h, dead larvae (motionless) were recorded, and the average percentage mortality was estimated [21]

**7. Statistical analysis**

Five replicates were done for all the treatments, and mean ± SE values were calculated. Larval mortality analysis was performed by using ANOVA and subsequent Duncan’s multiple range tests (p < 0.05). Also, Probit analysis was applied to determine the LC_50_ and LC_90_ values with their 95% confidence limits [22]. All statistical tests and analyses were achieved using SPSS (IBM SPSS Version 22.0).

**Results**

**GC-Ms analysis of the medicinal plants’ essential oils**

The GC-MS analysis of basil oil analysis demonstrated many components, but those with the highest concentrations were linalool, eucalyptol, and eugenol at 20, 12, and 7.91%, respectively (Supplementary Table 1). The analysis of cumin revealed a composition of 29.29% benzaldehyde, 4-(1-methylethyl), 17.1% à-Pinene and 15.42% 2-CAREN-10-AL (Supplementary Table 2). Clove oil contained 10.29% heptadecane, 11.34% Caryophyllene, 7.93% Hexadecane, 7.92% docosane, 7.91% dotriaconate, and 7.68% octadecane (Supplementary Table 3). Henna extract revealed 92.13% oleic acid, 5.42% n-Hexadecanoic acid, and 2.45% Hexadecanoic acid, methyl ester (Supplementary Table 4). The main components of ginger oil were 9,12-Octadecadienoic acid (Z,Z)- (23.83%), zingiberene (18.56%), α-curcumene (14.58%), á-sesquiphellandrene (12.85%), and á-Bisabolene (11.46%) (Supplementary Table 5).

**Characterization of the nanoemulsions prepared from the essential oils**

The hydrodynamic particle size of basil nanoemulsion was 215 nm with PDI 0.729. For cumin, the droplet size (DS) was 446.2 nm with PDI 0.047. The droplet size of clove nanoemulsion was 996.4 nm with PDI 0.224. The henna nanoemulsion droplet size was 273.9 with PDI 0.143. The droplet size of ginger nanoemulsion was 325.1 nm with PDI 0.059 (Table 7). The low PDI values reflected the homogenous size distribution of droplets in each essential nanoemulsion (Figs., 1 & 2).

**Table 1. Zeta sizer of the Nanoemulsion of each essential oil**

| Eos | Droplet size (nm) | PDI^*^ |
| --- | --- | --- |
| Basil | 215 | 0.729 |
| Cumin | 446.2 | 0.047 |
| Clove | 996.4 | 0.224 |
| Henna | 273.9 | 0.143 |
| Ginger | 325.1 | 0.059 |

* PDI = polydispersity index of nanoemulsions

Fig. 1. UV-vis spectrophotometer absorbance of different essential oils nanoemulsions

Fig. 2. Droplet size of different essential oils nanoemulsions

**Larvicidal activity of the used essential oils and their nanoemulsion forms**

Regarding the larvicidal potency of basil essential oils, the LC_50_ was 81.07 ug/mL and LC_90_ was 227.9ug/mL. The basil nanoemulsion, meanwhile, showed an LC_50_ of 65.19 ug/mL, and an LC_90_ of 193.3 ug/mL (Table 2). For cumin essential oils, it was 96.29 ug/mL and 64.50 ug/mL, respectively (Table 2). It was observed that in both of these cases the LC_50_ and LC_90_ values of the nanoemulsion forms were lower than the ordinary form. Turning to clove, the LC_50_ was 394 ug/mL, and LC_90_ was 1658 ug/mL, with henna the LC_50_ was 306 ug/mL and the LC_90_ was 1703 ug/mL for (Table 2). Ginger showed an LC_50_ and LC_90_ of 494 ug/mL and 1682 ug/mL, respectively. Moreover, it is noteworthy that the larvicidal effect of the essential oils and their nanoemulsion forms was concentration dependent. Basil and cumin essential oils achieved 100% larvicidal mortality at concentrations of 250, 500 and 1000 ug/mL. This insecticidal activity decreased to reach its minimal effect at the lowest concentration 31.25 ug/mL (Tables 3 & 4). In the case of clove, henna and ginger, however, the nanoemulsion had no additional effect beyond that of the essential oils (Table 2). Clove and ginger essential oils showed only weak larvicidal effects, such that only the highest concentration, 2000 ug/mL, caused 100% mortality of larvae (Table 5 & 6). For henna, meanwhile, concentrations of 1000 and 2000 ug/mL caused 100% mortality of larvae (Table 7).

**Table 2. LC50 and LC95 of the used essential oils after 24 hours of application**

| Treatments | LC50 (95 CI) | LC95 (95 CI) | Slope±SE |
| --- | --- | --- | --- |
| Basil ordinary form | 81.07 (64.2-102.6) | 227.9 (163.3-430.03) | 3.6±0.6 |
| Basil nanoemulsion | 65.19 (50.5-82.7) | 193.3(137.2-378.7) | 3.48±0.6 |
| Cuminum ordinary form | 96.29 (77.7-120.7) | 242.36(177.8-434.8) | 4.1±0.7 |
| Cuminum nanoemulsion | 64.5(48.3-83.4) | 222.9(151.5-489.09) | 3.05±0.6 |
| Clove ordinary form | 394(111-2096) | 1658(651-4882348) | 2.6±0.3 |
| Clove nanoemulsion | 379(198-810) | 1815(838-19457) | 2.4±0.3 |
| Henna ordinary form | 306(168-718) | 1703(724-31076) | 2.2±0.3 |
| Henna nanoemulsion | 288(153-712) | 1869(742-47639) | 2.02±0.3 |
| Ginger ordinary form | 494(388-636) | 1682(1171-3117) | 3.9±0.4 |
| Ginger nanoemulsion | 479(376-615) | 1623(1134-2935) | 3. 1±0.4 |

Table 3. Mean of larval mortalities after treatment with basil and its emulsion form after 24 hours application on the fourth larval stage of laboratory-reared *Culex pipiens*

| Form  Treatment | Basil Mean ±  Std. Error | Emulsion Basil Mean ±  Std. Error |
| --- | --- | --- |
| Control untreated | 4.80±.37^a^ | 4.80±.37^a^ |
| Deltamethrin (0.755ppm) | 100.0±.00^h^ | 100.0±.00^h^ |
| 1000 ug/mL | 100.00±.00^h^ | 100.00±.00^h^ |
| 500 ug/mL | 100.00±.00^h^ | 100.00±.00^h^ |
| 250 ug/mL | 100.00±.00^h^ | 100.00±.00^h^ |
| 125 ug/mL | 71.20±.37^f^ | 87.40±.24^g^ |
| 62.5 ug/mL | 29.80±.37^d^ | 37.80±.37^e^ |
| 31.25 ug/mL | 12.40±.24^b^ | 19.80±.37^c^ |

Superscript of the same letter in cells of the same column shows a non-significant effect in respect to the non-treated control. Superscript of different letters in cells of the same column shows a significant effect in respect to the non-treated control (*P* ≤ 0. 05).

Table 4. Mean of larval mortalities after treatment with cumin and its emulsion form after 24 hours application on the fourth larval stage of laboratory-reared *Culex pipiens*

| Form  Treatment | *Cuminum cyminum*  Mean ±  Std. Error | Emulsion  Mean ±  Std. Error |
| --- | --- | --- |
| Control untreated | 4.80 ±.37^a^ | 4.80 ±.37^a^ |
| Deltamethrin (0.755ppm) | 100.0±.00^h^ | 100.0±.00^h^ |
| 2000 ug | 100.00±.00^h^ | 100.00±.00^h^ |
| 1000 ug/mL | 100.00±.00^h^ | 100.00±.00^h^ |
| 500 ug/mL | 100.00±.00^h^ | 100.00±.00^h^ |
| 250 ug/mL | 100.0±.00^h^ | 100.0±.00^h^ |
| 125 ug/mL | 60.00±.44^d^ | 84.80±.37^e^ |
| 62.5 ug/mL | 20.00±.31^b^ | 34.80±.58^c^ |
| 31.2 ug/mL | 8.80±.37^a^ | 22.40±.40^b^ |

Superscript of the same letter in cells of the same column shows a non-significant effect in respect to the non-treated control. Superscript of different letters in cells of the same column shows a significant effect in respect to the non-treated control (*P* ≤ 0. 05).

Table 5. Mean of larval mortalities after treatment with Clove and its emulsion form after 24 hours application on the fourth larval stage of laboratory-reared *Culex pipiens*

| Form  Treatment | Clove Mean  Std. Error | Emulsion Mean  Std. Error |
| --- | --- | --- |
| Control untreated | 4.80±.37^a^ | 4.80±.37^a^ |
| Deltamethrin (0.755ppm) | 100.00±.00^e^ | 100.00±.00^e^ |
| 2000 ug | 100.00±.00e | 100.00±.00^e^ |
| 1000 ug/mL | 89.80±.37^d^ | 90.00±.31^d^ |
| 500 ug/mL | 54.80±.37^c^ | 54.80±.37^c^ |
| 250 ug/mL | 24.80±.37^b^ | 26.20±.37^b^ |
| 125 ug/mL | 7.40±.40^a^ | 8.40±.40^a^ |
| 62.5 ug/mL | 3.60±.24^a^ | 3.80±.20^a^ |
| 31.2 ug/mL | 2.60±.24^a^ | 3.80±.20^a^ |

Superscript of the same letter in cells of the same column shows a non-significant effect in respect to the non-treated control. Superscript of different letters in cells of the same column shows a significant effect in respect to the non-treated control (*P* ≤ 0. 05).

Table 6. Mean of larval mortalities after treatment with ginger and its emulsion form after 24 hours application on the fourth larval stage of laboratory-reared *Culex pipiens*

| Form Treatment | Ginger Mean  Std. Error | Emulsion Ginger Mean  Std. Error |
| --- | --- | --- |
| Control untreated | 4.80±.37^a^ | 4.80±.37^a^ |
| Deltamethrin (0.755ppm) | 100.00±.00^e^ | 100.00±.00^e^ |
| 2000 ug | 100.00±.00e | 100.00±.00^e^ |
| 1000 ug/mL | 88.60±.50^d^ | 90.00±.54^d^ |
| 500 ug/mL | 49.80±.37^c^ | 50.00±.31^c^ |
| 250 ug/mL | 22.40±.50^b^ | 24.80±.37^b^ |
| 125 ug/mL | 8.60±.24^a^ | 9.80±.20^a^ |
| 62.5 ug/mL | 3.60±.24^a^ | 4.80±.20^a^ |
| 31.2 ug/mL | 3.60±.24^a^ | 4.80±.20^a^ |

Superscript of the same letter in cells of the same column shows a non-significant effect in respect to the non-treated control. Superscript of different letters in cells of the same column shows a significant effect in respect to the non-treated control (*P* ≤ 0. 05).

Table 7. Mean of larval mortalities after treatment with henna and its emulsion form after 24 hours application on the fourth larval stage of laboratory-reared *Culex pipiens*

| Form Treatment | Henna Mean ±S.E | Emulsion Mean ±S.E |
| --- | --- | --- |
| Control untreated | 4.80±.37^a^ | 4.80±.37^a^ |
| Deltamethrin (0.755ppm) | 100.00±.00^e^ | 100.00±.00^e^ |
| 2000 ug | 100.00±.00e | 100.00±.00^e^ |
| 1000 ug/mL | 100.00±.00^e^ | 100.00±.00^e^ |
| 500 ug/mL | 64.80±.37^d^ | 65.00±.32^d^ |
| 250 ug/mL | 20.00±.32^c^ | 26.80±.37^c^ |
| 125 ug/mL | 15.20±.20^b^ | 19.80±.20^b^ |
| 62.5 ug/mL | 6.20±.37^a^ | 6.60±.51^a^ |
| 31.2 ug/mL | 3.60±.24^a^ | 4.80±.20^a^ |

Superscript of the same letter in cells of the same column shows a non-significant effect in respect to the non-treated control. Superscript of different letters in cells of the same column shows a significant effect in respect to the non-treated control (*P* ≤ 0. 05).
