# Supplementary material for: Using nanoemulsions of the essential oils of a selection of medicinal plants from Jazan, Saudi Arabia, as a green larvicidal against Culex pipiens
Source: PLoS One. 2022 May 23;17(5):e0267150. doi: 10.1371/journal.pone.0267150 (PMC9126372; doi:10.1371/journal.pone.0267150)
Supplement: S1 Table — (DOCX) [file pone.0267150.s002.docx]

**Supplementary Table 1. The phytochemical composition of Basil by GC-MS**

| peak | R.t* | Name | Area % | Molecular Weight | Molecular formula | MF** |
| --- | --- | --- | --- | --- | --- | --- |
| 1 | 4.22 | à-Pinene | 1.08 | 136 | C10H16 | 938 |
| 2 | 5.00 | (+)-BETA-PINEN | 1.42 | 136 | C10H16 | 932 |
| 3 | 6.26 | Eucalyptol | 12.45 | 154 | C10H18O | 906 |
| 4 | 6.85 | ç-Terpinene | 0.17 | 136 | C10H16 | 919 |
| 5 | 7.45 | 2-Carene | 0.30 | 136 | C10H16 | 839 |
| 6 | 9.27 | Linalool | 20.6 | 154 | C10H18O | 921 |
| 7 | 9.45 | Myroxide | 0.26 | 152 | C10H16O | 879 |
| 8 | 9.60 | Camphor | 1.30 | 152 | C10H16O | 919 |
| 9 | 9.67 | l-Menthone | 0.11 | 154 | C10H18O | 902 |
| 10 | 10.53 | Estragole | 6.67 | 148 | C10H12O | 919 |
| 11 | 11.50 | Carvone | 0.81 | 150 | C10H14O | 829 |
| 12 | 11.70 | 2,6-OCTADIEN-1-OL,  3,7-DIMETHYL-, (E)- | 0.23 | 154 | C10H18O | 841 |
| 13 | 12.20 | Bornyl acetate | 3.01 | 196 | C12H20O2 | 939 |
| 14 | 12.75 | 2-Propenoic acid, 3-phenyl-, methyl  Ester | 0.9 | 162 | C10H10O2 | 865 |
| 15 | 13.05 | ç-Elemene | 0.17 | 204 | C15H24 | 865 |
| 16 | 13.30 | alfa.-Copaene | 0.99 | 204 | C15H24 | 869 |
| 17 | 14.47 | Eugenol | 7.91 | 164 | C10H12O2 | 776 |
| 18 | 14.94 | Caryophyllene | 0.87 | 204 | C15H24 | 875 |
| 19 | 15.15 | Methyleugenol | 1.11 | 178 | C11H14O2 | 856 |
| 20 | 15.53 | trans-à-Bergamotene | 7.46 | 204 | C15H24 | 916 |
| 21 | 15.85 | Humulene | 2.95 | 204 | C15H24 | 926 |
| 22 | 15.99 | cis-Muurola-4(15),5-diene | 1.37 | 204 | C15H24 | 912 |
| 23 | 16.47 | Germacrene D | 6.96 | 204 | C15H24 | 920 |
| 24 | 16.67 | Aromandendrene | 4.09 | 204 | C15H24 | 907 |
| 25 | 17.16 | ç-Muurolene | 7.84 | 204 | C15H24 | 925 |
| 26 | 17.42 | CYCLOHEXENE,  4-(1,5-DIMETHYL-1,4-HEXADIE  NYL)-1-METHYL- | 1.55 | 204 | C15H24 | 910 |
| 27 | 17.85 | Nerolidol | 0.48 | 222 | C15H26O | 884 |
| 28 | 18.31 | SPATHULENOL | 0.63 | 220 | C15H24O | 909 |
| 29 | 18.93 | Epicubenol | 1.13 | 222 | C15H26O | 947 |
| 30 | 19.65 | tau.-Cadinol | 5.18 | 222 | C15H26O | 915 |
