# Supplementary material for: Using nanoemulsions of the essential oils of a selection of medicinal plants from Jazan, Saudi Arabia, as a green larvicidal against Culex pipiens
Source: PLoS One. 2022 May 23;17(5):e0267150. doi: 10.1371/journal.pone.0267150 (PMC9126372; doi:10.1371/journal.pone.0267150)
Supplement: S2 Table — (DOCX) [file pone.0267150.s003.docx]

**S Table 2. The phytochemical composition of cumin by GC-MS**

| Peak | R.t* | Name | Area % | Molecular Weight | Molecular formula | MF** |
| --- | --- | --- | --- | --- | --- | --- |
| 1 | 5.15 | à-Pinene | 17.1 | 136 | C10H16 | 929 |
| 2 | 6.31 | p-Mentha-1,5,8-triene | 2.47 | 134 | C10H14 | 812 |
| 3 | 7.79 | ç-Terpinene | 11.38 | 136 | C10H16 | 790 |
| 4 | 8.09 | Linalool | 0.35 | 154 | C10H18O | 854 |
| 5 | 9.77 | Terpinen-4-ol | 0.43 | 154 | C10H18O | 888 |
| 6 | 10.06 | 3-p-Menthen-7-al | 2.67 | 152 | C10H16O | 931 |
| 7 | 11.66 | Benzaldehyde, 4-(1-methylethyl)- | 29.39 | 148 | C10H12O | 915 |
| 8 | 13.30 | 2-CAREN-10-AL | 15.42 | 150 | C10H14O | 867 |
| 9 | 13.38 | p-Cymen-7-ol | 1.23 | 150 | C10H14O | 885 |
| 10 | 14.12 | Caryophyllene | 3.54 | 204 | C15H24 | 940 |
| 11 | 14.41 | á-Longipinene | 0.28 | 204 | C15H24 | 819 |
| 12 | 14.75 | ç-Elemene | 0.37 | 204 | C15H24 | 773 |
| 13 | 15.23 | cis-à-Bergamotene | 0.32 | 204 | C15H24 | 905 |
| 14 | 15.66 | cis-á-Farnesene | 2.05 | 204 | C15H24 | 905 |
| 15 | 16.28 | Aromandendrene | 8.07 | 204 | C15H24 | 883 |
| 16 | 16.97 | Cedrene | 0.71 | 204 | C15H24 | 911 |
| 17 | 17.87 | Caryophyllene oxide | 1 | 220 | C15H24O | 862 |
| 18 | 18.70 | Carotol | 2.74 | 222 | C15H26O | 938 |
| 19 | 20.07 | Isolongifolol | 0.48 | 222 | C15H26O | 761 |
