# Supplementary material for: Using nanoemulsions of the essential oils of a selection of medicinal plants from Jazan, Saudi Arabia, as a green larvicidal against Culex pipiens
Source: PLoS One. 2022 May 23;17(5):e0267150. doi: 10.1371/journal.pone.0267150 (PMC9126372; doi:10.1371/journal.pone.0267150)
Supplement: S3 Table — (DOCX) [file pone.0267150.s004.docx]

**S Table 3. The phytochemical composition of clove by GC-MS**

| peak | R.t* | Name | Area % | Molecular Weight | Molecular formula | MF** |
| --- | --- | --- | --- | --- | --- | --- |
| 1 | 13.70 | Copaene | 0.26 | 204 | C15H24 | 921 |
| 2 | 13.89 | Eugenol | 3.98 | 164 | C10H12O2 | 931 |
| 3 | 14.14 | Tetradecane | 1.76 | 198 | C14H30 | 963 |
| 4 | 14.74 | Caryophyllene | 11.34 | 204 | C15H24 | 929 |
| 5 | 15.14 | NONADECANOL | 0.52 | 284 | C19H40O | 806 |
| 6 | 15.42 | OCTADECANE, 1-CHLORO- | 6.06 | 288 | C18H37Cl | 732 |
| 7 | 16.25 | Pentadecane | 5.48 | 212 | C15H32 | 927 |
| 8 | 17.48 | Tetradecane, 2,6,10-trimethyl- | 5.35 | 240 | C17H36 | 839 |
| 9 | 18.27 | Hexadecane | 7.93 | 226 | C16H34 | 925 |
| 10 | 19.28 | 1-DOCOSANOL | 0.68 | 326 | C22H46O | 806 |
| 11 | 19.40 | DOCOSANE | 7.92 | 310 | C22H46 | 761 |
| 12 | 19.53 | DOTRIACONTANE | 7.91 | 450 | C32H66 | 785 |
| 13 | 20.18 | Heptadecane | 10.29 | 240 | C17H36 | 911 |
| 14 | 20.24 | TETRADECANE,  2,6,10-TRIMETHYL- | 2.90 | 240 | C17H36 | 869 |
| 15 | 21.95 | Octadecane | 7.68 | 254 | C18H38 | 926 |
| 16 | 23.62 | PENTACOSANE | 5.82 | 352 | C25H52 | 904 |
| 17 | 24.09 | Oxiraneundecanoic acid, 3-pentyl-, methyl ester, trans- | 1.21 | 312 | C19H36O3 | 766 |
| 18 | 25.21 | Eicosane | 4.71 | 282 | C20H42 | 926 |
| 19 | 28.24 | 11,14-Eicosadienoic acid, methyl ester | 3.31 | 322 | C21H38O2 | 815 |
| 20 | 28.35 | 9-OCTADECENOIC ACID (Z)- | 4.89 | 282 | C18H34O2 | 873 |
