# Supplementary material for: Using nanoemulsions of the essential oils of a selection of medicinal plants from Jazan, Saudi Arabia, as a green larvicidal against Culex pipiens
Source: PLoS One. 2022 May 23;17(5):e0267150. doi: 10.1371/journal.pone.0267150 (PMC9126372; doi:10.1371/journal.pone.0267150)
Supplement: S4 Table — (DOCX) [file pone.0267150.s005.docx]

**S Table 4. The phytochemical composition of henna by GC-MS**

| peak | R.t* | Name | Area % | Molecular Weight | Molecular formula | MF** |
| --- | --- | --- | --- | --- | --- | --- |
| 1 | 24.07 | Hexadecanoic acid, methyl ester | 2.45 | 270 | C17H34O2 | 886 |
| 2 | 25.59 | n-Hexadecanoic acid | 5.42 | 256 | C16H32O2 | 910 |
| 3 | 26.87 | Oleic Acid | 92.13 | 282 | C18H34O2 | 822 |
