# Supplementary material for: Using nanoemulsions of the essential oils of a selection of medicinal plants from Jazan, Saudi Arabia, as a green larvicidal against Culex pipiens
Source: PLoS One. 2022 May 23;17(5):e0267150. doi: 10.1371/journal.pone.0267150 (PMC9126372; doi:10.1371/journal.pone.0267150)
Supplement: S5 Table — (DOCX) [file pone.0267150.s006.docx]

**S Table 5. The phytochemical composition of zingber by GC-MS**

| peak | R.t* | Name | Area % | Molecular Weight | Molecular formula | MF** |
| --- | --- | --- | --- | --- | --- | --- |
| 1 | 4.61 | Camphene | 3.73 | 136 | C10H16 | 970 |
| 2 | 6.19 | cis-4-Thujanol | 6.09 | 154 | C10H18O | 843 |
| 3 | 9.41 | endo-Borneol | 1.52 | 154 | C10H18O | 932 |
| 4 | 13.69 | Copaene | 0.59 | 204 | C15H24 | 907 |
| 5 | 14.07 | β ELEMENE | 1.64 | 204 | C15H24 | 872 |
| 6 | 15.38 | cis-á-Farnesene | 1.18 | 204 | C15H24 | 904 |
| 7 | 16.04 | α-CURCUMENE | 14.58 | 202 | C15H22 | 968 |
| 8 | 16.29 | ZINGIBERENE | 18.56 | 204 | C15H24 | 907 |
| 9 | 16.52 | á-Bisabolene | 11.46 | 204 | C15H24 | 908 |
| 10 | 16.87 | á-SESQUIPHELLANDRENE | 12.85 | 204 | C15H24 | 956 |
| 11 | 24.06 | Hexadecanoic acid, methyl ester | 3.97 | 270 | C17H34O2 | 890 |
| 12 | 28.46 | 9,12-Octadecadienoic acid (Z,Z)- | 23.83 | 280 | C18H32O2 | 873 |
